# High Pressure Balloon Dilatation of Primary Obstructive Megaureter in Children: A Multicenter Study

**DOI:** 10.3389/fped.2018.00329

**Published:** 2018-10-31

**Authors:** Ibtissam Kassite, Mariette Renaux Petel, Yann Chaussy, Emilie Eyssartier, Khalid Alzahrani, Caroline Sczwarc, Thierry Villemagne, Hubert Lardy, Karim Braik, Aurélien Binet

**Affiliations:** ^1^Department of Pediatric Surgery, University Teaching Hospital of Tours, Gatien de Clocheville Hospital, Tours, France; ^2^Department of Pediatric Surgery, University Teaching Hospital of Rouen, Charles Nicolle Hospital, Rouen, France; ^3^Department of Pediatric Surgery, University Teaching Hospital of Besançon, Jean Minjoz Hospital, Besancon, France; ^4^Department of Pediatric Surgery, University Teaching Hospital of Angers, Angers, France

**Keywords:** ureteral diseases, ureteroscopy, pediatric surgery, hydronephrosis, pyelonephritis

## Abstract

**Aim of the Study:** We described the initial experience of four referral centers in the treatment of primary obstructive megaureter (POM) in children, by high-pressure balloon dilatation (HPBD) of the ureterovesical junction with double JJ stenting. We managed a retrospective multicenter study to assess its effectiveness in long-term.

**Methods:** We reviewed the medical records of all children who underwent HPBD for POM that require surgical treatment from May 2012 to December 2017 in four different institutions. The primary outcome measured was ureterohydronephrosis (UHN) and its degree of improvement after the procedure. Secondary outcomes were postoperative complications and resolution of preoperative symptomatology.

**Main Results:** Forty-two ureters underwent HPBD for POM in 33 children, with a median age of 14.7 months – (range: 3 months −15 years). Ureterohydronephrosis improves in 86% of ureters after one endoscopic treatment. Three cases required a second HPBD. Four patients required surgical treatment for worsening of UHN after endoscopic treatment. The post-operative complication rate was 50% (21 ureters). In 13 cases (61%), they were related to double J stent. The median follow-up was 24 months (2 months −5 years) and all patients were symptom-free.

**Conclusion:** We reported the first multicenter study and the largest series of children treated with HPBD, with an overall success rate of 92%. Endoscopic treatment can be a definitive treatment of POM since it avoided reimplantation in 90% of cases. Complications are mainly due to double J stent.

## Introduction

In the majority of cases, primary obstructive megaureter (POM) improves or disappears spontaneously without compromising renal function (1, 2). Thereby, conservative management is currently used for initial approach for POM.

These past two decades, surgical management of POM was revised. For decades reimplantation with ureteral tapering has been an established treatment for progressive or persistent POM associated with significant obstruction and/or infection (3). However, the disproportion between the size of the dilated ureter and the size of the bladder especially in children of <1 year makes this procedure challenging and exposes to bladder dysfunction (4).

In the midst of minimally invasive surgery, the search for less invasive procedures for treating POM has resulted in various surgical temporizing or definitive options.

Endoscopic balloon dilatation has been shown to be a feasible, and less invasive procedure with success rates ranging from 67 to 95% (5–8). Unlike open surgery, the endoscopic approach avoids traumatizing the bladder and the distal ureteral blood supply is left intact (9). Furthermore, reimplantation can still be performed if endoscopic treatment fails.

The objective of this study is to describe the initial experience of 4 referral centers in the treatment of POM by high-pressure balloon dilatation (HPBD) of the ureterovesical junction with double JJ stenting and to assess its effectiveness in the long-term.

## Materials and methods

We conducted a retrospective multicenter study. We reviewed the medical records of all children who underwent HPBD for POM that require surgical treatment from May 2012 to December 2017 in four different institutions (Tours, Rouen, Angers and Besançon).

Approval was received from the institutional review board.

Data were abstracted into a retrospective database for analysis.

Patient age, gender, presenting symptoms and follow up were analyzed.

POM diagnosis was based on the following criteria: dilation of the distal ureter by more than 5 mm, absence of VUR or bladder outlet obstruction after voiding cystourethrogram (VCUG).

### Preoperative management

All patients were evaluated by renal ultrasound (RUS), VCUG and dimercaptosuccinic acid (DMSA) or MAG3 scintigraphy. The diameter of the renal pelvis, parenchymal thinning and ureteral dilation were evaluated by RUS. VCUG was performed to rule out VUR and bladder outlet obstruction.

The prophylactic antibiotics since the diagnostic of POM was not systematic. The attitude varies depending on the institution.

The indications for surgery were a combination of clinical, ultrasonographic (US) and scintigraphic findings. Recurrent febrile urinary tract infections (UTI) or breakthrough febrile UTI instead of prophylaxis were clinical criteria indicating surgery in these patients. US criteria were increased hydroureteronephrosis or parenchymal thinning. Impairment of more than 10% of differential renal function (DRF) or (DRF) <40% were the criteria on scintigraphic findings indicating surgery. The exclusion criteria for balloon dilatation of the ureterovesical junction were infants with other urinary tract anomalies or neurological urinary disorders or VUR.

### Technique

As previously described (10), under general anesthesia, and a single dose of antibiotic prophylaxis, a cystoscope with a working channel was used. The UVJ was bypassed with a Terumo® flexible guidewire (0.018 or 0.035), introduced through the renal pelvis. The UVJ was dilated with a 6.0-mm Cook® balloon catheter filled with radiologic contrast agent, which was passed over the guidewire. The UVJ was dilated at 17 atmospheres (14–26) for 10 min, under direct fluoroscopic control, until the narrow ring disappeared.

### Postoperative management

After dilatation, a 6 × 16–24 cm double J stent was left in place (pyelo-vesical) and was removed 3 months after surgery. No bladder catheter was retained. The patient was discharged after 24–48 h. Follow-up consisted of US scans at 3, 6, and 12 months after surgery and VCUG was performed only in cases of UTI. A second HPBD was performed if obstructive manifestations persisted. Ureteral reimplantation was performed if the second HPBD was unsuccessful in a symptomatic patient.

### Outcomes

Primary outcome measured was postoperative improvement in degree of hydroureteronephrosis. Secondary outcomes related to development of postoperative complications (bleeding, infection, urinoma, stent migration) and resolution of preoperative symptomatology. Success of the operation was judged at follow up and was principally based on ultrasound findings at 3, 6, and 12 months. It is defined as improvement or stabilization of renal function, elimination of the UTIs episodes, and reduction of the degree of hydrouteronephrosis. VCUG was performed only in cases of UTI.

### Statistical analysis

The previous collected data were described by median, with ranges for quantitative variables, and frequencies with percentages for qualitative variables. Qualitative data were analyzed by a Fisher's exact test and the Wilcoxon rank sum test were used to compare quantitative data. Epi InfoTM 7.2 software was used for all analysis. The significance threshold was set at 0.05.

## Results

From May 2012 to December 2017, a total of 33 patients and 42 ureters were treated with the endoscopic balloon dilatation for POM. There were 27 boys and 6 girls, with a median age at surgery of 14.7 months (range: 3 months −15 years). Sixteen cases were left sided, 26 were right sided and nine were bilateral. Eleven cases were diagnosed prenatally. Nine patients had more than 1 criterion to indicate surgery (Table 1).

**Table 1:** Patients demographics and preoperative characteristics (*N* = 33).

Number of patients	33
Number of ureters	42
Age at surgery	14.7 months (3 months −15 years)
**Side**	
Bilateral	9
Unilateral	24
Left	16
Right	26
**Indication for surgery**	
Worsening hydronephrosis	6
UTIs	13
Decrease renal function	6
Others	8
More than 1 indication	9
**Preoperative diameter**	
Pelvis [median (range)]	17 (5–43)
Distal ureter [median (range)]	15 (5–30)

*UTIs, urinary tract infections*.

Ureterohydronephrosis improves in 86% of ureters after one endoscopic treatment. There were intra-operative difficulties in ureter catheterization in 3 cases, and ureteral stent migration occured in 2 cases. Three patients needed repeat dilatation because of hydronephrosis worsening after double J stent removal (Table 2). After endoscopic treatment, 13 cases had a febrile UTI during stenting period, which were related to double J stent in 11 cases. In the two other cases, VCUG was done with no signs of VUR. There were 13 (61%) post-operative complications related to Double J stent (11 UTIs and two stent encrustation) and two intra-operative stent migration. Post-operative complications categorized by the Clavien–Dindo classification showed 11 cases of grade II and 10 cases of grade IIIb (Table 2).

**Table 2:** Surgical outcomes of HPBD for POM.

Success rate after first dilatation (%)	86
**Results**	
Improvement/disappearance of HN	16/19
Stable HN	1
Worsening of HN	6
Secondary surgery	10
Re-dilatation	3
Reimplantation	4
Others	3
Follow up (median)	24 months (2 months−5 years)
**Complications**	
Intraoperative	5
Postoperative	21
-Grade II (UTIs)	11
-Grade IIIb (six worsening of HN, two stent encrustation, two UTIs)	10
Postoperative VUR	0

*VUR, vesico ureteral reflux; HN, hydronephrosis*.

Statistical analysis revealed significant differences before and after surgery in the diameter of the pelvis and the ureter (Table 3).

**Table 3:** Renal US finding after endoscopic treatment.

	**Preoperative**	**12 months postoperative**	***P*-value (Wilcoxon test)**
Mean renal pelvis diameter (mm)	16.7 (0–43)	6 (0–25)	<0.05
Mean distal ureter diameter (mm)	15.3 (2–30)	5.3 (0–16)	<0.05

In four cases, the surgeons attempted HPBD without ureteral stenting. There were no need for reintervention and patients were symptom-free at last check-up.

Patients who received two treatments had no more risk of failure, or of postoperative vesicoureteral reflux or ureteral reimplantation than those who received only 1 treatment (Table 4).

**Table 4:** Comparison between 1 and 2 endoscopic treatment.

	**1 HPBD**	**2 HPBD**	***P*-value (Fisher's test)**
Success	36/42	2/3	NS
Postoperative VUR	0	0	NS
Ureteral reimplantation	3/42	1/3	NS

## Discussion

Since the first report of endoscopic balloon dilation for POM in children in 1998 by Angulo (7), Several publications described this approach as feasible, safe and a less-invasive for very young patients than traditional open ureteral reimplantation with tapering (Table 5).

**Table 5:** Published series on endoscopic balloon dilatation for POM in children.

**Center/Country**	**Author**	**Type of study**	**Single/Multicenter**	**Study duration (m)**	**Sam-ple size**	**Age (range)**	**Preop pelvis/ure-ter diameter (mm)**	**Follow up**	**Postop pelvis/ure-ter diameter (mm)**	**Duration of stenting**	**Success rate after first dilata-tion (%)**	**Secondary treatment**	**HUN improve-ment rate (%)**	**Complica-tions**	**Postop VUR rate (%)**
Cadiz (Spain)	Angulo et al. (7)	Retrospec-tive	single center	16	11	NA	NA	NA	NA	NA	54	5 redilatations	100	NA	18
Barcelo-na (Spain)	Angerri et al. (6)	Retrospec-tive	Single center	49	7	12 (5–34) m	NA/NA	31 (12–56) m	NA/NA	2 months	71	1 redilatation	85	1 UTI	14
Barcelo-na (Spain)	García-Aparicio et al. (11)	Retrospec-tive	Single center	25	13	7 (4–24) m	27/14	25 (12–36) m	0/5	2 months	46	5 redilatations 3 reimplanta-tions	85	2 UTI	15
Philadel-phia (USA)	Christman et al. (9)	Prospecti-ve	Single center	NA	17	7 (3–12) y	NA/NA	3.2 (2–6.5) y	NA/NA	4–6 weeks	71	0	71	2 urolithiasis	0
Rome (Italy)	Torino et al. (12)	Retrospec-tive	Single center	13	5	8 (6–12) m	NA/NA	24 (16–30) m	NA/NA	6–8 weeks	100	0	100	0	0
Madrid (Spain)	Romero et al. (13)	Retrospec-tive	Single center	85	29	4 m	18/14	47 (18–104) m	7/8	4–6 weeks	69	7 reimplanta-tions 2 redilatations	76	5 UTI	17
Rome (Italy)	Capozza et al. (14)	Retrospec-tive	Single center	50	12	8 (6–12) m	NA/18	21 (2–44) m	NA/10	6–8 weeks	58	3 reimplanta-tions 3 cutting balloon	83	0	0
Barcelo-na (Spain)	García-Aparicio et al. (15)	Retrospec-tive	Single center	66	20	14 (3–103) m	24/15	49 (14–80)	6/6.6	2 months	60	5 redilatations 3 reimplanta-tions	86	4 UTI 1 hematuria	27
Barcelo-na (Spain)	Bujons et al. (16)	Retrospec-tive	Single center	116	19	17 (1–44) m	21/19	6.9 (3.9–13.3) y	3/3	2 months	90	1 redilatation	95	2 lithiasis 2 UTI	5
Tours (France)	Kassite et al. (10)	Retrospec-tive	Single center	36	12	14 (9–84) m	18/15	12.5 (6–30) m	5/7	3 months	83	2 redilatations 1 reimplanta-tion	92	7 UTI	0
Spain	Casal Beloy et al. (17)	Retrospective	Single center	93	13	9 (2–24) m	20/15	10.3 (4.7–12.2) y	11/4	4–6 weeks	100	0	NA	4 UTI	NA

*M, months; Y, years; UTI, urinary tract infections*.

Our data appears to be compatible with other studies that have reported short series with good results in the short, medium and long term. All the studies were retrospective and single- centered (Table 5).

Christman et al. reported outcomes of endoscopic incision and balloon dilation with double stenting for the treatment of primary obstructive megaureter in children (9). They performed laser incision followed by balloon dilation if the narrowed segment was 2–3 cm, with a success rate of 71% and no need for reintervention. They believed it is the relative motion of the two stents with peristalsis that allows for a lasting expansion of the dilated adynamic segment at the ureterovesical junction and for prevention of synechia formation (9).

Capozza et al. performed a cutting balloon ureterotomy (CBU) in cases with persistent stenotic ring after balloon dilatation (14). The overall success rate was 83% and no complications were reported. Casal Beloy et al. published a success rate of 100%, without re-interventions or secondary treatment (17). They excluded all patients with ureter >25 mm which explains this high success rate.

There is concern that dilating the ureterovesical junction could result in secondary VUR (14). In France, it's not in our practice to realize a VCUG systematically in the absence of symptoms. The incidence of post-operative VUR varies between 5 and 27% according to the literature (15, 16). Not all reports of endoscopic management of POM have routinely included postoperative VCUG (Table 5). Garcia-aparicio et al. reported that VUR is a transient condition after HPBD (15). They observed a relationship between postoperative VUR and paraureteral diverticula (*p* > 0.05), since patients with any paraureteral pathology, such as diverticula will have poor detrusor backing, and HPBD increases it. They also found a relationship between post-operative VUR and bilateral POM (*p* > 0.05).

We can't conclude about our rate of post-operative VUR because we didn't perform VCUG systematically. VCUG appears to be an invasive investigation and we think it's useless to perform it routinely if patients remain asymptomatic and continue to show normal renal function without hydronephrosis or UTI.

In our series, complications were mainly related to ureteral stenting. This raised the question of the real need for ureteral stenting after HPBD. Routine stent placement has been questioned for a long time, especially in relation to balloon dilatation and ureteroscopy for urolithiasis (18). The use of a stent is recommended only for the short period required to prevent the obstruction and renal failure due to edema, epithelial hyperplasia, or inflammatory cell reaction (19). Moreover, some authors have recently suggested that the ureteral stent is not necessary in uncomplicated procedures (20, 21). That's why, the last 4 cases of this series were dilated without ureteral stenting and no complication ocurred at follow-up.

The period of time that the stent should be maintained is also controversial. In experimental studies, ureteral oedema and consequent obstruction have been observed for more than 72–96 h after ureteral dilation (22). In a study comparing two groups of patients with and without stent, the authors did not find any difference in terms of postoperative complications (18).

## Conclusion

This first multicenter study showed that endoscopic balloon dilation for POM is a safe procedure, less invasive than reimplantation, and shows good outcomes on long-term follow-up. Endoscopic treatment can be a definitive treatment of POM since it avoided reimplantation in 90% of cases. However, complications were mainly related to double J stent, raising the question of the real need for ureteral stenting after HPBD. Also, prospective studies are required to demonstrate definitively the real benefits of this approach and whether there is a real need for double J stenting.

## Author contributions

IK and AB: Conception and design. IK, MR, YC, EE, and KA: Data acquisition. IK, CS, and TV: Data analysis and interpretation, drafting the manuscript. HL, KB, and AB: Critical revision of the manuscript for scientific and factual content. IK and AB: Statistical analysis. HL and AB: Supervision.

### Conflict of interest statement

The authors declare that the research was conducted in the absence of any commercial or financial relationships that could be construed as a potential conflict of interest.
